# Sawfly Sex Pheromones: Analysis of Their Impact on Pine Odor Attractive to Egg Parasitoids

**DOI:** 10.1007/s10886-024-01547-1

**Published:** 2024-09-17

**Authors:** Asifur Rahman-Soad, Ludwig Skuras, Andreas Reinecke, Martti Varama, Monika Hilker

**Affiliations:** 1 Applied Zoology/Animal Ecology, Institute of Biology, Freie Universität Berlin, Berlin, Germany; 2 Natural Resources Institute Finland, Helsinki, Finland

**Keywords:** Pine, Sawfly, Egg parasitoids, Semiochemicals, Tritrophic interaction

## Abstract

*Pinus sylvestris* trees are known to efficiently defend themselves against eggs of the herbivorous sawfly *Diprion pini*. Their direct defense against eggs is primable by prior exposure to the sex pheromones of this species and their indirect defense involves attraction of egg parasitoids by egg-induced pine needle odor. But it is unknown whether exposure of pine to *D. pini* sex pheromones also affects pine indirect defense against sawfly eggs. In this study, we investigated the influence of exposure of *P. sylvestris* trees to the sex pheromones of *D. pini* on indirect defense mediated by egg parasitoids. Behavioral assays with *Closterocerus ruforum*, a key parasitoid of sawfly eggs, revealed no significant attraction to odor from egg-free pines pre-exposed to pheromones. Chemical analyses of odor from egg-free pines showed no pheromone-induced change in the emission rates of the known key terpenoids promoting parasitoid attraction. Further comparative analyses of odor from egg-laden pines pre-exposed to the sex pheromones and of odor from egg-laden pines unexposed to pheromones neither revealed significant differences in the emission rates of terpenoids relevant for parasitoid attraction. The results suggest that a pheromone-induced or pheromone-primed, egg-induced pine indirect defense seems to be redundant in addition to the known pheromone-primable pine direct defense against the eggs and the known egg-inducible indirect defense.

**Supplementary Information:**

The online version contains supplementary material available at 10.1007/s10886-024-01547-1.

## Introduction

Plants emit volatile organic compounds (VOCs) that influence a myriad of interactions in trophic networks (Dicke 2016; Turlings and Erb 2018; Kessler et al. 2023; Kutty and Mishra 2023; Schuman 2023). Of these interactions, the attraction of parasitoids to plant VOCs emitted in response to insect herbivory or egg deposition has been intensively studied with respect to so-called plant indirect anti-herbivore defenses (Vet and Dicke 1992; Hilker and Fatouros 2015; Aljbory and Chen 2018; Ali et al. 2023; Gómez-Cabezas et al. 2023).

Certain plant volatile carboxylic acid esters, such as (*Z*)-3-hexenyl acetate, methyl jasmonate and methyl salicylate, have been recognized as inducers or priming agents of plant defenses against herbivorous insects (Engelberth et al. 2004; Frost et al. 2008; Kegge et al. 2013; Mageroy et al. 2020). Carboxylic acid esters are also prominently released as sex pheromones by a wide variety of herbivorous insect species (Francke and Schulz 2010; Naka and Fujii 2020). Notably, pheromones ranging from simple alkyl esters to more complex diesters have been identified across various species (Tabata et al. 2012; Tabata and Ichiki 2017; Meier et al. 2020). While a wide range of studies have investigated how plant VOCs influence the emission and perception of insect sex pheromones (Reddy and Guerrero 2004; Arx et al. 2012; Xu et al. 2017; Borrero-Echeverry et al. 2018; Hoffmann et al. 2020), little is known about how insect sex pheromones affect the emission of plant VOCs, which in turn may influence the behavior of herbivorous insects and their antagonists.

The sex pheromone components released by female *Diprion pini* sawflies are carboxylic acid esters, specifically (*2S*,*3R*,*7R*)-3,7-dimethyl-2-tridecanyl acetate and propionate (Bergström et al. 1995; Anderbrant et al. 2005). These chemical signals possess the remarkable ability to traverse great distances, effectively serving as beacons that can allure males (Anderbrant et al. 1995, 2005). Previous studies have demonstrated that the pheromones of *D. pini* are attractive to the eulophid egg parasitoid *Closterocerus ruforum* (Hilker et al. 2000), a species specialized on eggs of pine-infesting diprionid sawflies (Pschorn-Walcher and Eichhorn 1973; Eichhorn and Pschorn‐Walcher 1976). The pheromones of the sawfly may serve as an early indication for the egg parasitoid that *D. pini* is present, potentially indicating the availability of host eggs. Moreover, *C. ruforum* females are attracted by the egg-induced pine odor. *Pinus sylvestris* twigs carrying three-day-old *D. pini* eggs on their needles emit enhanced quantities of (*E*)-β-farnesene, which, in combination with four other non-induced pine terpenoids is attractive to the parasitoid (Mumm et al. 2003; Beyaert et al. 2010).

Interestingly, *D. pini* sex pheromones have been shown to prime pine trees’ direct defenses against sawfly eggs. Sawfly eggs laid on pheromone-exposed pine needles suffer lower survival rates compared to eggs on unexposed trees (Bittner et al. 2019). This priming activity of the *D. pini* pheromones on pine direct defenses against sawfly eggs raised the question of whether the pheromones also impact pine indirect defenses involving egg parasitoid attraction.

Here, we studied (i) whether exposure of egg-free pine trees to *D. pini* pheromones can induce *P. sylvestris* VOCs attractive to these parasitoids and/or (ii) whether the pheromones can prime the egg-induced emission of (*E*)-β-farnesene, which attracts the egg parasitoid *C. ruforum* to egg-laden pine when combined with four other non-induced pine terpenoids.

We addressed the question on the inductive capability of *D. pini* pheromones on parasitoid-attracting pine VOCs by comparing, first, the parasitoids’ behavioral response to odor from (egg-free) pheromone-exposed and unexposed pine trees. Furthermore, we chemically analyzed whether pheromone-exposed pine differs from unexposed pine by the emission rates of (*E*)-β-farnesene and the other pine VOCs that, in combination with the egg-induced quantities of (*E*)-β-farnesene, provide an odor attractive to the parasitoids (Beyaert et al. 2010). In addition, we investigated whether the exposure of pine to *D. pini* sex pheromones further enhances (primes) the egg-induced emission of (*E*)-β-farnesene and/or affects the emission of the other four pine VOCs relevant for attraction of *C. ruforum.*

By exploring the attraction of egg parasitoids to pheromone-exposed pine and the potential effects of the pheromones on pine VOCs, this study aims to contribute to our understanding of the complex network of chemical communication in the tripartite interactions among plants, herbivorous insects and parasitoids.

## Methods and Materials

### Plants

For the experiments, three-year-old *P. sylvestris* trees (40–60 cm height, 2–4 cm stem Ø) were purchased from a local tree nursery (Baumschule Stackelitz GmbH and Co. KG, 06868 Coswig / OT Stackelitz, Germany). Each tree grew individually in pots filled with Classic T potting soil (Einheitserde^®^, a mixture of peat and clay; *N* = 340 mg·L^− 1^, P_2_O_5_ = 260 mg·L^− 1^, K_2_O = 330 mg·L^− 1^). Prior to the experiments, the trees were kept in a greenhouse under long-day conditions (20 °C, 18:6 h, L:D cycle). One week before starting the experiments, the trees were transferred to a climate chamber, where they could acclimate to the abiotic experimental conditions (20 °C, 18:6 h, L:D cycle, 70% RH, 100 µmol photons m^− 2^·s^− 1^). Each potted tree was kept in a transparent Plexiglas^®^ cylinder (14.1 L, 80 cm height, 15 cm Ø). The cylinder was equipped with an inlet for charcoal-filtered air at the bottom (250 mL·min^− 1^) and an air outlet on top (250 mL·min^− 1^). Thus, the trees inside the cylinders were exposed to only charcoal-filtered air. The pots were wrapped with polyethylene terephthalate (PET) bags covering the pots and soil, thus preventing VOCs released from the soil or the roots from affecting aboveground pine responses.

### Insects

*Diprion pini* were reared under laboratory conditions in a climate chamber (20 °C, 18:6 h, L:D cycle, 70% RH, 100 µmol photons m^− 2^·s^− 1^) following the protocol from Eichhorn (1976). Females laid eggs onto the needles of *P. sylvestris* branches collected from a forest southwest of Berlin. The eggs are laid in a row of about 10–15 eggs onto a pine needle. Each pine branch was provided with tap water and kept inside a transparent Plexiglas^®^ cylinder (14.9 L, 50 cm height, 19.5 cm Ø), which was closed on top with a gauze lid. Larvae hatched from eggs about 12 to 14 d after egg deposition and started feeding on pine needles. Pupation began approximately three weeks after larval hatching. Cocoons were collected and stored at 4 °C. To initiate the emergence of adults, the cocoons were transferred back to the rearing climate chamber five days before use for the experiments. Following emergence, the adults were transferred to a separate climate chamber (10 °C, 18:6 h, L:D cycle, 70% RH, 100 µmol photons m^− 2^·s^− 1^) until required for the experiments.

The eulophid egg parasitoid *C. ruforum* was obtained from parasitized eggs of a close relative of *D. pini*, i.e. the pine sawfly *Neodiprion sertifer.* The parasitized eggs were collected in a Finnish forest, sent to our Berlin laboratory, where they were stored in Petri dishes at 5 °C for 14 days at maximum. To initiate parasitoid emergence, we transferred the Petri dishes containing needles with parasitized eggs to 20 °C (18:6 h, L:D cycle, 70% RH). Emergence of adult parasitic wasps from the sawfly eggs typically started 8 to 10 days later. Upon emergence from host eggs, adult parasitoids were individually placed in a small Petri dish (3 cm Ø) and stored at 10 °C (18:6 h, L:D cycle, 70% RH). For feeding, GIZEH^®^ filter papers (3 × 15 mm) soaked in a 15% aqueous honey solution were added to each dish. For bioassay preparation, females were allowed to mate at 20 °C. Therefore, a male was placed into each dish with a female for 24 h. Thereafter, female parasitoids were separated from the males and kept for an additional period of 24 h (‘lag phase’) under the conditions described for storage (see above). After the lag phase, the bioassays were conducted.

### Treatment of Pine

Pine trees were exposed to synthetic male-attracting sex pheromones of *D. pini* females, specifically (*2S*,*3R*,*7R*)-3,7-dimethyl-2-tridecanyl acetate and propionate, supplied by the laboratory of Olle Anderbrant, Lund University, Sweden. We used the same pheromone concentrations as those known to prime pine direct defense against *D. pini* eggs; these concentrations are comparable to those which pine trees are exposed to during *D. pini* mass outbreaks (Bittner et al. 2019). Thus, the pheromone esters were each dissolved in hexane at a concentration of 50 ng·µL^− 1^. For treatment of the trees, 100 µL of the pheromone solution (50 µL (*2S*,*3R*,*7R*)-3,7-dimethyl-2-tridecanyl acetate + 50 µL (*2S*,*3R*,*7R*)-3,7-dimethyl-2-tridecanyl propionate) was applied to a cotton wool pad (5.6 cm Ø, 0.4 cm thickness) as the dispenser. To allow for solvent evaporation, the cotton pads were left under a fume hood for 30 min before being exposed to the trees. After solvent evaporation, the cotton pad with pheromones was placed into a Plexiglas^®^ cylinder (14.1 L, 80 cm height, 15 cm Ø) with a pine tree for 24 h.

Because the pheromones were dissolved in hexane, a solvent control with 100 µL hexane only was also included. A pad was treated with only 100 µL hexane, placed under a fume hood for 30 min, and thereafter placed into a cylinder with a pine tree for 24 h.

A second control (blank) was provided by adding an untreated cotton pad without any solvent to a cylinder with a pine tree.

First, egg-free pine trees were subjected to the above-described treatments to investigate whether exposure of pine to the pheromones (or hexane) can induce a change in pine odor. We used *n* = 5–6 replicates per treatment.

In addition, to study whether exposure of pine to the pheromones (or hexane) affects the release of the known egg-induced pine volatiles, the above-described treatments were followed by *D. pini* egg depositions for 24–72 h, until each tree had received three to four egg rows on its needles. The successful egg laying was monitored at timely intervals. The insects were removed from the trees after the targeted number of egg masses had been laid. For this combined treatment by exposure to volatiles and subsequent oviposition, we used *n* = 5–8 replicates per treatment.

### Olfactometer Bioassay with Egg Parasitoids

Olfactometer bioassays with parasitoids were conducted using a four-field olfactometer. The design of the olfactometer was similar to the setup described by Hilker et al. (2002). In short, odor from four sources flowed into four olfactometer fields at a rate of 155 ml·min^− 1^. To determine whether the egg parasitoid is attracted by odor released by pheromone-exposed pine, one field was ventilated with odor from a pheromone-treated pine, and the opposite field was ventilated either with odor from a hexane-treated pine or an untreated control pine. The remaining two fields were considered buffer fields, ventilated with charcoal-filtered air only. The parasitoids were exposed to odor from pheromone-exposed (or hexane-exposed) pine right after the end of the 24 h-exposure period, and also one and two days later.

After a parasitoid female was placed into the center of the olfactometer, its behavior was recorded for a period of 10 min using the software Observer 3.0 (Noldus, Wageningen, The Netherlands). We recorded for how long the parasitoids were actively moving around (walking) in the olfactometer field supplied with odor from pheromone-exposed pine compared to the opposite field and also in the buffer fields. The majority of the tested parasitoid females was actively moving in these olfactometer fields. We calculated the relative walking activity as percentage active walking time from total observation time (10 min; 100%) (for detailed information on the activity of the parasitoids in the fields supplied with odor from differently treated pine and in the buffer fields, see Supplemental Table S1). When offering the parasitoids the odor from a pheromone-treated tree and from an untreated (blank) pine in the opposing field, we tested in total *n* = 9 parasitoids/time point and *n =* 3 trees/treatment. When offering the odor from a pheromone-treated tree and a hexane-treated tree in the opposing field, we tested in total *n* = 18 parasitoids/time point and *n =* 6 trees/treatment.

### Collection of Pine Volatiles

Pine VOCs were collected from pheromone-treated, hexane-treated and untreated (only cotton pad) *P. sylvestris* trees. One set of these trees was without sawfly egg depositions, the other set received egg depositions after the treatment with pheromones (or hexane). The collection of pine volatiles was always conducted between 10 am and 11 am, during the first third of the daylight phase in the climate chamber (20 °C, 18:6 h, L:D cycle, 70% RH, 100 µmol photons m^− 2^·s^− 1^).

To investigate possible changes in emission rates of volatiles in the course of time post treatments, we collected pine VOCs at different time points. The headspace of each egg-free tree was sampled at four time points: directly (0 h) post exposure, and 24 h, 48 h, and finally 72 h post pheromone or hexane exposure. The headspace of egg-laden trees was also collected at four time points, i.e. 24 h, 48 h, 72 h, and 96 h after the end of the oviposition period.

The VOCs emitted from the differently treated pine trees were collected on Tenax^®^ TA filters. Prior to VOC collection, the filters were conditioned at 280 °C. Filters were integrated into the cylinder’s outlet airflow, and collection was conducted at a rate of 100 mL·min^− 1^ for 1 h with an inflow of charcoal-filtered air maintained at 200 mL·min^− 1^ from the bottom. After the trapping of volatiles, they were always analyzed on the same day.

### Chemical Analysis of Pine Terpenes

The headspace volatiles from pine trees were analyzed using gas chromatography-mass spectrometry (7890 A and 5975 C VL MSD, Agilent, Waldbronn, Germany). Trapped pine VOCs were desorbed from the filter in a thermal desorption unit (TDU) (GERSTEL, Mülheim, Germany). For later quantification of the pine VOCs, 2.5 µL methyl octanoate (50 ng·µL^− 1^) were injected as internal standard (IS) into each Tenax^®^ TA filter with pine VOCs; this was done just prior to introducing the filter into the TDU. The TDU started at 30 °C and then heated up at a rate of 100 °C min^− 1^ until it reached 290 °C, where it was held for 3 min. Subsequently, the VOCs were concentrated in a Programmable Temperature Vaporization (PTV) type inlet (CIS 4, Gerstel) at -50 °C. The temperature of the cryotrap then increased from − 50 °C to 290 °C at a rate of 12 °C sec^− 1^ to transfer the VOCs to the column. The VOCs were transferred in splitless mode to an HP5-MS column UI capillary column (30 m x 250 μm x 0.25 μm) with helium as a carrier gas at a flow rate of 1 mL·min^− 1^. The front PTV inlet for helium was set to solvent vent mode, with a purge flow to split vent set at 5 mL·min^− 1^ starting at 0.01 min. The temperature programming of the oven included two stages: initially set at 40 °C for 5 min, the temperature increased at a rate of 5 °C·min^− 1^ to reach 260 °C without any holding period, followed by a sharper increase at 60 °C·min^− 1^ up to 300 °C, which was then sustained for 10 min. Mass spectra of the VOCs were obtained by electron impact ionization at 70 eV (scan mode range 33 to 350 *m/z*).

The analysis of the trapped pine volatiles focused on those five terpenoids that are known to be crucial for the positive (electrophysiological and behavioral) response of the egg parasitoid *C. ruforum* to egg-induced pine odor (Beyaert et al. 2010). These compounds — β-phellandrene, (*E/Z*)-β-ocimene, β-caryophyllene, α-humulene, and (*E*)-β-farnesene — are collectively effective in attracting *C. ruforum* when mixed in the same ratios as emitted from egg-laden pine. While the emission of (*E*)-β-farnesene is induced by *D. pini* egg deposition (Mumm et al. 2003; Mäntyla et al. 2018), the emission of the four other compounds was so far not found to be affected by *D. pini* egg deposition, but necessary to be combined with the egg-induced emission rate of (*E*)-β-farnesene for attraction of *C. ruforum* (Beyaert et al. 2010).

For identification of these terpenoids, authentic standards of each compound were acquired from the following sources: (*E/Z*)-β-ocimene and (*E*)-β-farnesene from Sigma-Aldrich (Taufkirchen, Germany), β-phellandrene from Biomol (Hamburg, Germany), β-caryophyllene and α-humulene from Fluka (Fluka / Fisher Scientifics, Schwerte, Germany) and methyl octanoate from TCI (Eschborn, Germany). Pine VOCs were identified by comparison of their mass spectra and retentions indices with those of authentic standards.

For quantification, standard samples with known concentrations (1, 2.5, 5, 10, 25, 50 and 100 ng·µL^− 1^) were analyzed under identical GC-MS conditions as the pine tree headspace samples by injecting an aliquot of 2.5 µL into a Tenax^®^ TA filter. We measured the peak areas of the compounds in total ion current chromatograms (TICs). To cope with potential measurement variability over time (Noonan et al. 2018), peak areas of the standard samples were normalized against the internal standard (methyl octanoate), which had been injected into the Tenax^®^ TA filter as well (injection volume: 2.5 µL; concentration: 50 ng·µL^− 1^). Dose-response curves (concentration standard sample vs. normalized peak area) were generated using linear regression models. Peak areas of pine VOCs were also normalized against the IS peak area. Some pine VOCs were detected with relative peak areas smaller than the ones measured at the lowest concentration of the standard runs. For example, the mean of the smallest measured relative areas of α-humulene as standard sample (1 ng·µL^− 1^) was 0.11 (11% of the measured area of the IS peak). Therefore, if a pine VOC sample had a relative area less than this, it was not approximated with the curve provided by the linear regression analysis, but rather with a curve that goes from the lowest measured point through the origin.

On the HP5-MS column, β-phellandrene was not separated from limonene and was therefore calculated by the following formula as described by Mumm et al. (2003):

$$\:X=\frac{{A}_{68}\cdot\frac{{C}_{93}}{{B}_{68}}}{{A}_{68}\cdot\frac{{C}_{93}}{{B}_{93}}+({A}_{93}-{A}_{68}\cdot\frac{{C}_{93}}{{B}_{68}}\cdot\frac{{B}_{93}}{{B}_{68}})}Y=1-X$$

The variables stand for the following. Y: portion of β-phellandrene in total ion current chromatogram (TIC)-peak of β-phellandrene + limonene, X: portion of limonene in the TIC-peak of β-phellandrene + limonene, B_68_: portion of mass 68 of the total ion yield of a limonene pure sample (%), A_68_: peak area of ion trace 68; A_93_: peak area of ion trace 93, B_93_: portion of mass 93 of the total ion yield of a limonene pure sample (%), and C_93_: portion of mass 93 of the total ion yield of a β-phellandrene pure sample (%).

### Statistical Analysis

Graphing and data analysis were performed using Prism version 10.2.1 (GraphPad Software). Initially, data normality was assessed using the Shapiro-Wilk method, followed by appropriate statistical tests. P-values greater than 0.05 were considered not significant (ns), while p-values below 0.05 were deemed significant.

For the behavioral response data of the egg parasitoid, the Wilcoxon matched-pairs signed rank test was used to compare parasitoid activity levels in different olfactometer fields.

For the emission rates of pine key terpenoids, differences among the treatment groups were determined using the Kruskal-Wallis test by comparing emission rates from (i) untreated, hexane-exposed, pheromone-exposed pine without egg deposition among each other and (ii) from untreated, hexane-exposed, pheromone-exposed pine with egg deposition among each other. Additionally, a Mann-Whitney *U* test was performed to further evaluate the impact of *D. pini* egg deposition on emission rates of the analyzed terpenoids from the young trees used here (comparison of odor from egg-free pine and odor from egg-laden pine, both without exposure to pheromones).

## Results

### Olfactometer Bioassay with Egg Parasitoids

**Fig. 1:**
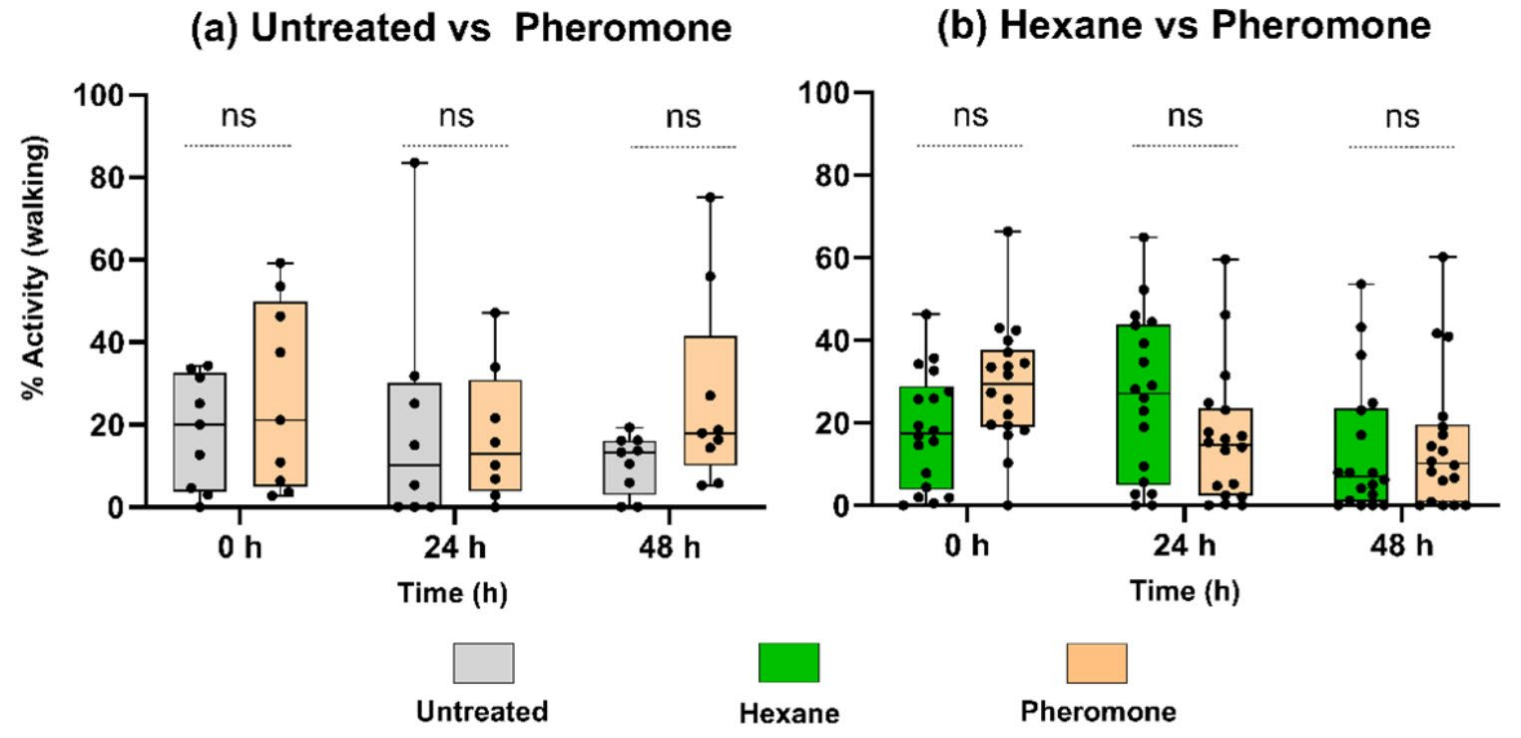
Behavioral response of the egg parasitoid *Closterocerus ruforum* to odor from *Pinus sylvestris* trees 0, 24, and 48 h post exposure to either *Diprion pini* pheromones or hexane or were left untreated. The activity level of the parasitoids was measured by recording the time they spent walking in the olfactometer field that was supplied with the odor from pheromone-exposed trees and in the opposite field with odor from untreated trees (**a**) or trees exposed to hexane (**b**). The total observation lasted for 10 min. Data expressed as percentage active walking time from total observation time (100%). The whiskers represent the minimum and maximum values, and the middle line inside boxes denotes the median, (**a**) *n* = 9 parasitoids/time point; *n* = 3 trees/treatment, (**b**) *n* = 18 parasitoids/time point; *n* = 6 trees/treatment. Statistical analysis: Wilcoxon matched-pairs signed rank test; ns not significant (*p* > 0.05)

The olfactometer bioassays showed that the egg parasitoid *C. ruforum* is not attracted by odor from pheromone-exposed pine when compared to odor from untreated pine (Fig. 1a) or to odor from hexane-exposed pine (Fig. 1b). The time during which the parasitoid females walked around in the olfactometer field supplied with odor from pheromone-exposed pine did not significantly differ from the time in the opposing field supplied with odor from untreated or hexane-exposed pine (For details on parasitoid activity in fields with odor from treated pine and buffer fields, see Supplemental Table S1). This lack of preference for pheromone-exposed pine was consistent across the different time points post exposure.

### Chemical Analysis of Pine Terpenes

**Fig. 2:**
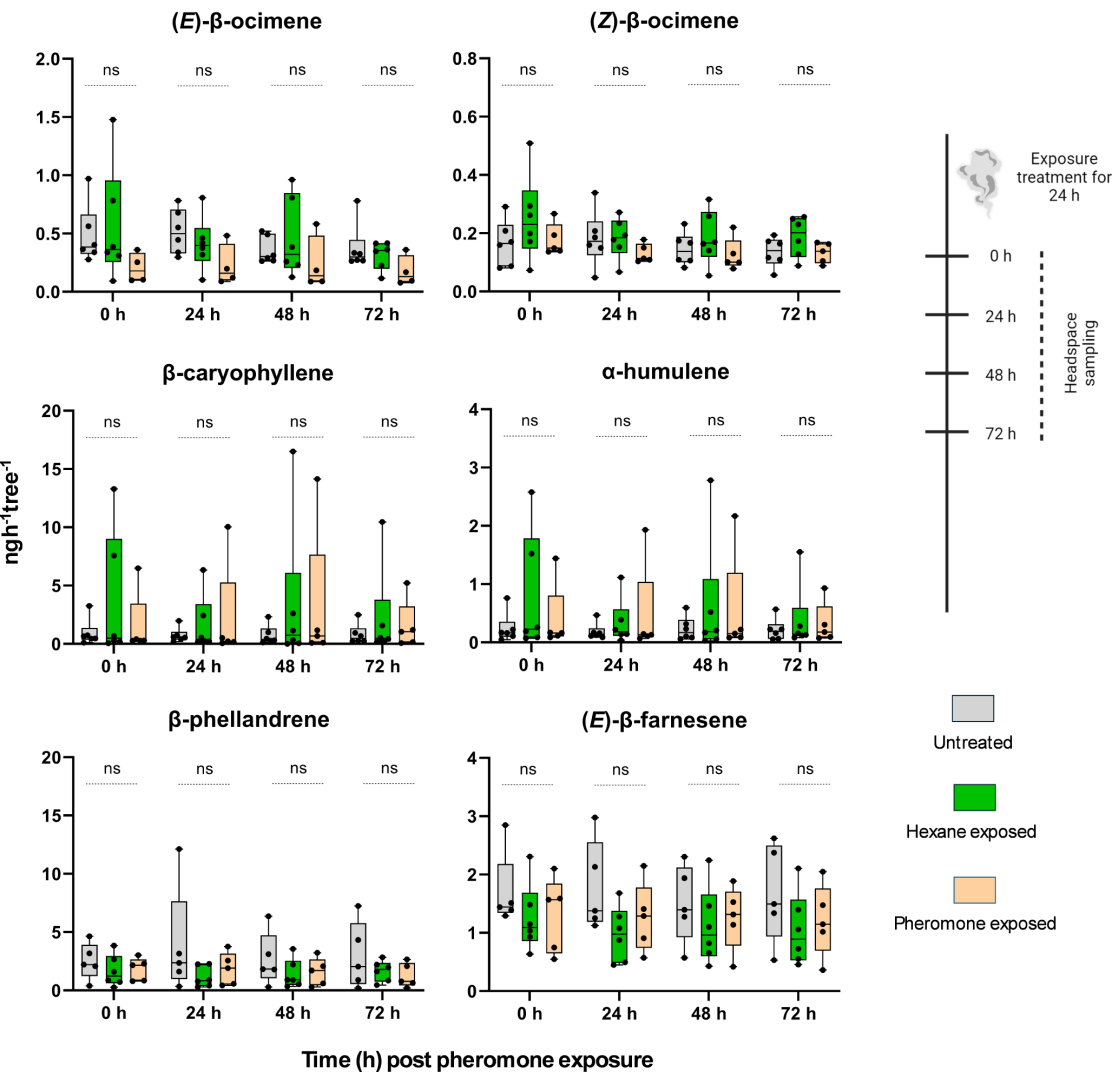
GC-MS analyses of the odor from differently treated, egg-free *Pinus sylvestris* trees. Emission rates of pine key terpenoids that are known to attract the egg parasitoid *Closterocerus ruforum* were recorded. Trees were left untreated or were exposed to hexane or *Diprion pini* pheromones. Headspace samples were collected repeatedly from each tree at four different time intervals after the end of pine treatments: 0, 24, 48, 72 h. The whiskers represent the minimum and maximum values, and the middle line inside the boxes denotes the median (*n* = 5–6 trees per treatment). Statistical analysis: Kruskal-Wallis test; ns not significant (*p* > 0.05)

The headspace of differently treated *P. sylvestris* trees was analyzed with respect to the emission rates of five key volatile components, which are known to attract the egg parasitoid *C. ruforum* (Beyaert et al. 2010). Among these five components, emission rates of both stereoisomers (*E* and *Z*) of β-ocimene were included. Headspace samples were collected directly post treatment (0 h) and at intervals of 24, 48, and 72 h post treatment from trees exposed to *D. pini* pheromones, hexane, or from trees that were left untreated. The GC-MS analysis revealed no significant differences in the emission rates of these substances across the treatments at any time sampling point (Fig. 2, Supplemental Table S2).

**Fig. 3:**
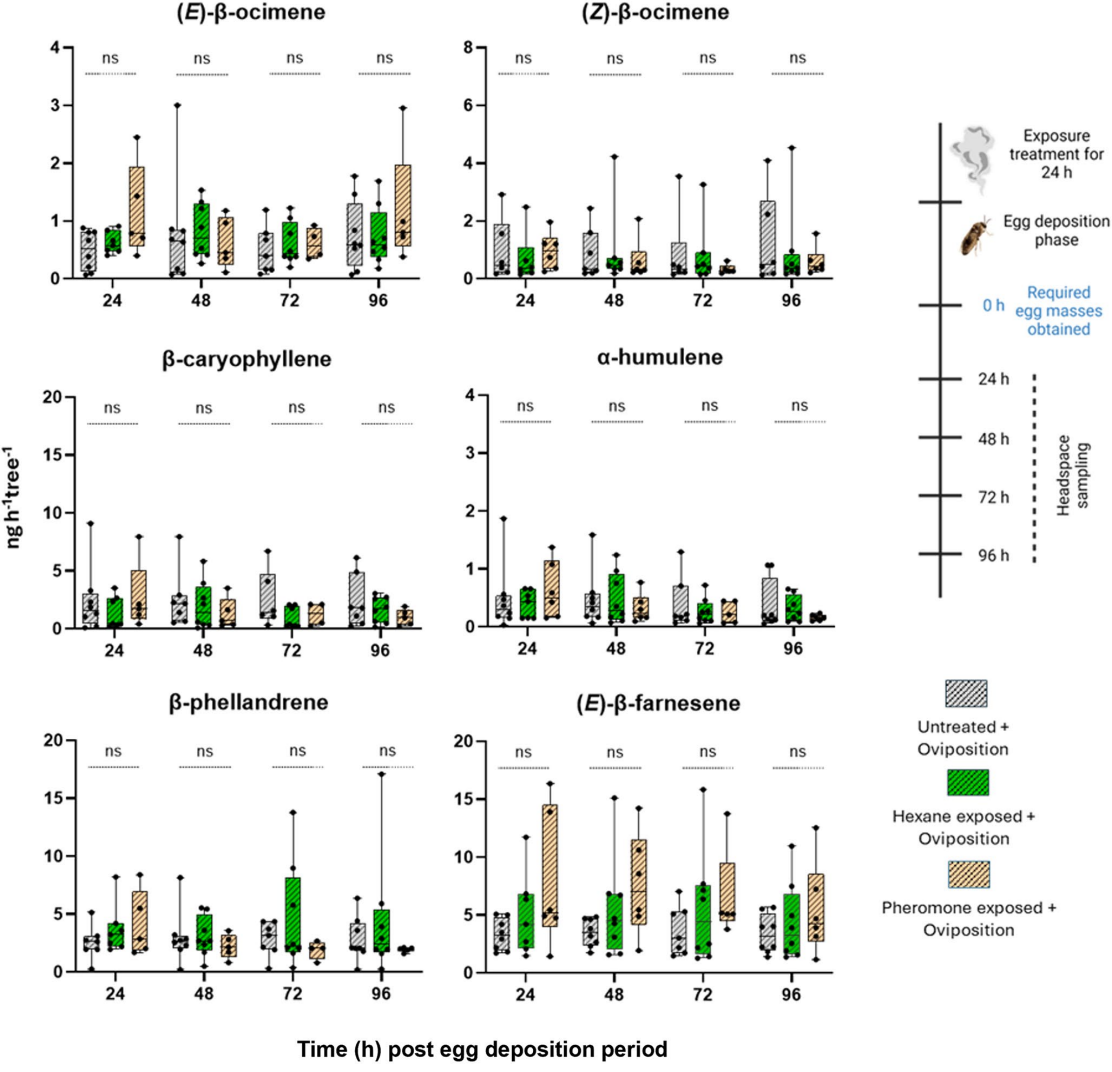
GC-MS analysis of differently treated *Pinus sylvestris* trees laden with *Diprion pini* eggs. Emission rates of pine key terpenoids that are known to attract the egg parasitoid *Closterocerus ruforum* were recorded. Trees were left untreated or exposed to hexane or *D. pini* pheromones, followed by egg deposition by *D. pini* females. Headspace samples were collected repeatedly from each tree at four different time intervals after the end of the egg deposition period: 24, 48, 72 h, and 96 h. The whiskers represent the minimum and maximum values, and the middle line inside the boxes denotes the median (*n* = 5–8 trees per treatment). Statistical analysis: Kruskal-Wallis test; ns not significant (*p* > 0.05)

To test whether the exposure of pine to *D. pini* pheromones affects the emission rate of egg-induced pine, we analyzed in a follow-up study the emission rates from *P. sylvestris* first exposed to pheromones (or hexane) and subsequently subjected to *D. pini* egg deposition. Again, we focused our analysis on those five key terpenoids that are known to be relevant for attraction of the egg parasitoid *C. ruforum.* The emission rates of (*E)*-β-farnesene and (*E)*-β-ocimene released from previously pheromone-exposed, egg-laden pine were by trend higher than those released from control trees (Fig. 3). However, our analyses revealed no significant differences in the emission rates of these compounds when comparing the pheromone-treated, egg-laden pine with the respective egg-laden control pine trees (treated with hexane or left untreated) by a Kruskal-Wallis test (Fig. 3, Supplemental Table S2).

To test whether the young pine trees used in our study respond to *D. pini* egg deposition as known for branches of mature pine trees (Mumm et al. 2003; Mäntyla et al. 2018), we compared the emission rates of the analyzed terpenoids from untreated pine and from pine with egg deposition. In accordance with the response of mature trees to *D. pini* egg deposition, the egg-laden, young pine trees emitted significantly higher quantities of (*E*)-β-farnesene than egg-free pine (Supplemental Table S3). Interestingly, the egg-laden pine trees studied here emitted also higher quantities of (*Z*)-β-ocimene than egg-free ones, whereas mature trees did not show a significantly egg-induced emission of this terpenoid compound (Supplemental Table S3).

## Discussion

Previous research showed that exposure of *P. sylvestris* to *D. pini* sex pheromones primes pine direct defenses against sawfly eggs and leads to decreased egg survival rates (Bittner et al. 2019). Here, we investigated whether exposure of pine to the sawfly’s pheromones also impacts pine indirect defenses against the eggs. Studies of the behavioral responses of the egg parasitoid *C. ruforum* revealed that exposure of pine to the pheromones did not induce a pine odor that is attractive to this antagonist of the sawfly eggs. Our comparative chemical analyses of the headspace of pheromone-exposed and control pines revealed no significant differences in the emission rates of those terpenoids that are known to be relevant for the attraction of the parasitoid (Beyaert et al. 2010). We furthermore, investigated whether exposure of pine to the pheromones can prime the egg-inducible emission rate of (*E*)*-*β-farnesene, which is known to attract the parasitoid when combined with four further pine terpenoids that are not egg-induced (Beyaert et al. 2010). However, comparative chemical analyses of egg-laden pines exposed to pheromones and those unexposed showed no significant difference in the emission rate of (*E*)*-*β-farnesene.

The egg parasitoid *C. ruforum* is known to be attracted to the odor from pine induced by host egg deposition, but not to the odor from untreated, egg-free host plants (Hilker et al. 2002). Our finding that the egg parasitoids were not attracted to pheromone-exposed pines (Fig. 1) matches the lack of any notable pheromone-induced change in the emission rate of those pine volatiles relevant for attraction of *C. ruforum* (Fig. 2). Furthermore, the lack of attraction of *C. ruforum* by odor emitted from pheromone-exposed pine supports a previous study by Bittner et al. (2019), which showed that young *P. sylvestris* trees that had been exposed to *D. pini* pheromones as done in our study do not release any pheromonal traces after a 24-h exposure period. In accordance with this previous finding, the egg parasitoid *C. ruforum*, which is known to be attracted by *D. pini* pheromones (Hilker et al. 2000), did not prefer the odor from pine exposed to *D. pini* pheromone to odor from unexposed pine.

Since we tested parasitoids collected in forests from eggs of *Neodiprion sertifer*, but not from *D. pini* eggs, it cannot be excluded that the lack of attraction by odor from pine exposed to *D. pini* pheromone was due to local adaptation of the tested individuals to their natal host species, its pheromone and the impact of the *N. sertifer* pheromone on the host plant. The pheromone of *N. sertifer* has been identified as (2*S*, 3*S*, 7*S*)-3,7-dimethyl-2-pentadecanol esterified with acetic acid (Jewett et al. 1976; Wassgren et al. 1992), thus differing from the *D. pini* pheromone by its stereochemistry and the chain length of the alcohol component of the ester. Especially in parthenogenetically reproducing parasitoids, local adaptation to the environment may facilitate maintenance of within-species genetic variance and support successful development in host species of different quality (Godfray 1994; Harvey et al. 2012; Hopper et al. 2019; Harrison et al., 2022). In our previous studies, which showed attraction of *C. ruforum* to *D. pini* sex pheromones (Hilker et al. 2000) and to pine odor induced by *D. pini* eggs (Hilker et al. 2002; Schröder et al. 2008), we also used individuals collected from *N. sertifer* eggs at the same locations in Finland as the parasitoids used in our current study. These previous results showed that those previously collected *C. ruforum* individuals, which developed in *N. sertifer* eggs, can respond to infochemicals associated with *D. pini.*

Trees exposed to pheromones of herbivorous insects face the challenge of responding to cues that do not indicate the precise location of the attacker since a pheromone plume may widely disperse within the tree canopy. The attraction of *C. ruforum* to *D. pini* sex pheromones (Hilker et al. 2000) might help the parasitoid in habitat location when orientating towards the odor source, while the attraction to egg-induced pine odor (Hilker et al. 2002) can help the parasitoid locate egg-laden needles. If exposure of egg-free pine to *D. pini* sex pheromones were to induce an odor attractive to the parasitoid, this might interfere with these known attractive odors, and thus might be no beneficial pine response to the sex pheromones of its attacker.

However, if exposure of pine to *D. pini* sex pheromones would prime the emission rate of egg-inducible (*E*)*-*β-farnesene, such a priming effect could enhance the attraction of *C. ruforum* and guide more parasitoids to egg-laden pine needles. Interestingly, the data show that shortly after the end of the oviposition period, the emission rates of especially (*E*)*-*β-ocimene and (*E*)*-*β-farnesene tended to be higher in previously pheromone-exposed, egg-laden pine than in egg-laden pine without prior pheromone exposure. However, our chemical analyses found no evidence for a significant priming effect of *D. pini* pheromones on the egg-inducible emission rate of (*E*)-β-farnesene. Thus far, there is no indication that an increase in the emission rate of (*E*)*-*β-ocimene is relevant for attraction of *C. ruforum.* This egg parasitoid species is highly sensitive to (*E*)*-*β-farnesene and is attracted by it only when combined with four other pine terpenes – among them (*E*)*-*β-ocimene – which are not inducible by *D. pini* egg deposition per se (Mumm et al. 2003; Beyaert et al. 2010). Future studies should elucidate the ecological relevance of the enhanced emission rate of (*E*)*-*β-ocimene in pheromone-exposed, egg-laden pine compared to pheromone-exposed, egg-free pine.

Our study corroborates previous findings (Mumm et al. 2003; Mäntyla et al. 2018), which showed that *D. pini* egg deposition induces the emission of (*E*)-β-farnesene, a key terpene for the attraction of the egg parasitoid *C. ruforum.* In previous studies, attraction of this parasitoid by egg-induced pine odor and the enhanced emission rate of (*E*)-β-farnesene were found 72 h after *D. pini* egg deposition (Hilker et al. 2002; Mumm et al. 2003; Schröder et al. 2008). In contrast to these previous studies, we found (i) that also (*Z*)-β-ocimene was induced by the sawfly egg deposition and (ii) that (*E*)*-*β-farnesene is not only induced 72 h post egg deposition, but already earlier after 24 h (Supplemental Table S3). These differences may be due to different developmental stages of pine used for our study and the previous ones. Unlike previous studies that analyzed the response of pine twigs from mature trees to sawfly egg deposition, we sampled volatiles from egg-laden, three-year-old pine saplings. Our findings suggest that pine saplings respond more swiftly to egg deposition by *D. pini* and accelerate the release of VOCs, which are crucial for attracting antagonists of the eggs. This finding is supported by previous molecular studies of *P. sylvestris* laden with *D. pini* eggs. A recent transcriptomic analysis of pine saplings laden with eggs of *D. pini* showed upregulation of genes involved in terpene biosynthesis as soon as one hour post egg deposition (Hundacker et al. 2024). In contrast, a previous analysis of twigs taken from mature trees revealed that transcription rates of the sesquiterpene synthases *PsTPS* 1 and 2 (encoding (*E*)-β-caryophyllene and α-humulene for *PsTPS* 1, and 1(10),5-germacradiene-4-ol for *PsTPS* 2) were only enhanced 72 h after *D. pini* egg deposition (Köpke et al. 2008).

Taken together, no significant effects of the exposure of pine to *D. pini* sex pheromones on pine indirect defenses against the sawfly eggs were detected in our study. In contrast, *D. pini* sex pheromones were previously shown to affect (prime) pine direct defenses against the eggs, thus providing evidence for the responsiveness of pine to these insect volatiles (Bittner et al. 2019). Plant indirect and direct defenses have often been discussed and studied regarding trade-offs due to resource allocation costs (Cipollini et al. 2003; Agrawal and Fishbein 2006; Agrawal 2011). An indirect, pheromone-elicited defense of pine against the infestation with *D. pini* eggs might be too costly, as it would be in addition to the known egg-inducible, indirect defense response Hilker et al. 2002; Bittner et al. 2019) and the pheromone-primable, direct defense against eggs (Bittner et al. 2019). We suggest that the attraction of egg parasitoids to *D. pini* sex pheromones and egg-induced pine odor renders a pheromone-induced or pheromone-primed, egg-induced ‘cry for help’ (Dicke et al. 1990) redundant. The benefits of a defensive plant response to insect infestation are expected to, at least, balance or, at best outweigh the costs for the defense (Pearse et al. 2020). If the attraction of *C. ruforum* by *D. pini* sex pheromones and egg-induced pine odor provides sufficient protection of pine trees, any further investment of pine into additional signaling might represent an unnecessary energy expenditure.

## Electronic Supplementary Material

Below is the link to the electronic supplementary material.

Supplementary Material 1

## Data Availability

Data will be made available upon request.
