# Supplementary material for: Sawfly Sex Pheromones: Analysis of Their Impact on Pine Odor Attractive to Egg Parasitoids
Source: J Chem Ecol. 2024 Sep 17;50(11):620–30. doi: 10.1007/s10886-024-01547-1 (PMC11543748; doi:10.1007/s10886-024-01547-1)
Supplement: Supplementary file 1 — Supplementary Material 1 [file 10886_2024_1547_MOESM1_ESM.docx]

**Supplement**

**Sawfly Sex Pheromones: Analysis of Their Impact on Pine Odor Attractive to Egg Parasitoids**

ASIFUR RAHMAN-SOAD^1^, LUDWIG SKURAS^1^, ANDREAS REINECKE^1^, MARTTI VARAMA^2^, AND MONIKA HILKER^1*^

*^1^Applied Zoology/Animal Ecology, Institute of Biology, Freie Universität Berlin, Berlin, Germany
^2^Natural Resources Institute Finland, Helsinki, Finland*

Asifur Rahman-Soad: ORCID: <https://orcid.org/0000-0001-7387-5391>

Andreas Reinecke: ORCID: <https://orcid.org/0000-0002-9336-7324>

Monika Hilker: ORCID: <http://orcid.org/0000-0001-7519-7395>

*Corresponding author:

Monika Hilker, Applied Zoology/Animal Ecology, Institute of Biology, Freie Universität Berlin, Berlin, Germany, Email: monika.hilker@fu-berlin.de ORCID: <http://orcid.org/0000-0001-7519-7395>

Supplemental Table S1: Detailed information about the walking activity of the egg parasitoid *Closterocerus ruforum* in four-field olfactometer assays (compare Fig. 1, main manuscript). The walking activity was recorded when testing the parasitoids’ response to odor from *Pinus sylvestris* trees 0, 24, and 48 h post exposure to either *Diprion pini* pheromones or hexane or were left untreated. Tested: Odor from pheromone-exposed tree versus untreated tree (left table); odor from pheromone-exposed tree versus hexane-treated tree (right table); for each test: two buffer fields. Data show (i) relative walking time (%) of total observation period (10 min = 100%) for each tested parasitoid individual in each olfactometer field, (ii) the “total (sum)” of the relative walking times for each parasitoid individual in all four olfactometer fields, and (iii) the overall mean walking activity ± SE of all parasitoids when studying their response to “pheromone vs untreated” and when studying their response to “pheromone vs hexane” (calculated from “total (sum)” data).

Supplemental Table S2: Statistical details (Kruskal-Wallis tests) on the comparison of odor from *Pinus sylvestris* subjected to different treatments: (i) without *Diprion pini* egg deposition, involving exposure to either *D. pini* pheromones (P) or to hexane (H) or were left untreated (U) and (ii) with sawfly egg deposition (E) that followed the same treatments as described for (i). Headspace samples were collected repeatedly from each tree at four different time intervals after the end of pine treatments without egg deposition (0, 24, 48, 72 h) and after the pine treatments with egg deposition (24, 48, 72, 96 h). The emission rates (means ± SE, ng x h^-1^ x tree^-1^) of pine key terpenoids that are known to attract the egg parasitoid *Closterocerus ruforum* are given as well as the *p* values of the statistical comparisons.

| Compound | Time point (h) | Without egg deposition | | | | Time point (h) | With egg deposition | | | |
| --- | --- | --- | --- | --- | --- | --- | --- | --- | --- | --- |
|  |  | **Untreated (U)** | **Hexane**  **(H)** | **Pheromone (P)** | **Group comparison (U vs H vs P)**  **P-values** |  | **Untreated + Oviposition (U+E)** | **Hexane + Oviposition (H+E)** | **Pheromone + Oviposition (P+E)** | **Group comparison (U+E vs H+E vs P+E) P-values** |
| (*E*)-β-ocimene | **0** | 0.48 ± 0.10 | 0.56 ± 0.20 | 0.20 ± 0.06 | 0.14 | **24** | 0.48 ± 0.12 | 0.62 ± 0.07 | 1.15 ± 0.36 | 0.35 |
|  | **24** | 0.51 ± 0.07 | 0.41 ± 0.09 | 0.22 ± 0.08 | 0.19 | **48** | 0.79 ± 0.33 | 0.82 ± 0.17 | 0.61 ± 0.19 | 0.94 |
|  | **48** | 0.35 ± 0.04 | 0.46 ± 0.13 | 0.23 ± 0.11 | 0.30 | **72** | 0.49 ± 0.15 | 0.60 ± 0.13 | 0.6 ± 0.13 | 0.89 |
|  | **72** | 0.37 ± 0.08 | 0.31 ± 0.04 | 0.17 ± 0.06 | 0.19 | **96** | 0.74 ± 0.21 | 0.73 ± 0.18 | 1.17 ± 0.45 | 0.75 |
| (*Z*)-β-ocimene | **0** | 0.16 ± 0.03 | 0.25 ± 0.06 | 0.17 ± 0.02 | 0.45 | **24** | 0.97 ± 0.44 | 0.69 ± 0.36 | 0.95 ± 0.26 | 0.29 |
|  | **24** | 0.18 ± 0.03 | 0.18 ± 0.02 | 0.13 ± 0.01 | 0.29 | **48** | 0.84 ± 0.32 | 0.97 ± 0.54 | 0.62 ± 0.29 | 0.61 |
|  | **48** | 0.14 ± 0.02 | 0.18 ± 0.03 | 0.12 ± 0.02 | 0.40 | **72** | 0.84 ± 0.32 | 0.82 ± 0.41 | 0.32 ± 0.07 | 0.71 |
|  | **72** | 0.13 ± 0.02 | 0.18 ± 0.02 | 0.13 ± 0.01 | 0.22 | **96** | 1.26 ± 0.64 | 0.92 ± 0.52 | 0.59 ± 0.20 | 0.53 |
| β-caryophyllene | **0** | 0.94 ± 0.47 | 3.67 ± 2.26 | 1.54 ± 1.23 | 0.76 | **24** | 2.39 ± 1.02 | 1.39 ± 0.52 | 2.68 ± 1.34 | 0.85 |
|  | **24** | 0.73 ± 0.25 | 1.65 ± 1.00 | 2.19 ± 1.96 | 0.69 | **48** | 2.55 ± 0.96 | 1.98 ± 0.73 | 1.29 ± 0.60 | 0.83 |
|  | **48** | 0.74 ± 0.33 | 3.44 ± 2.64 | 3.23 ± 2.73 | 0.99 | **72** | 2.48 ± 0.99 | 0.90 ± 0.31 | 1.24 ± 0.50 | 0.53 |
|  | **72** | 0.81 ± 0.35 | 2.22 ± 1.66 | 1.54 ± 0.94 | >0.99 | **96** | 2.35 ± 0.86 | 1.62 ± 0.39 | 0.97 ± 0.31 | 0.64 |
| α-humulene | **0** | 0.24 ± 0.10 | 0.78 ± 0.42 | 0.39 ± 0.26 | 0.74 | **24** | 0.48 ± 0.20 | 0.39 ± 0.08 | 0.63 ± 0.20 | 0.61 |
|  | **24** | 0.18 ± 0.05 | 0.34 ± 0.16 | 0.47 ± 0.36 | 0.78 | **48** | 0.47 ± 0.17 | 0.48 ± 0.15 | 0.32 ± 0.10 | 0.88 |
|  | **48** | 0.23 ± 0.08 | 0.62 ± 0.43 | 0.54 ± 0.40 | 1.00 | **72** | 0.39 ± 0.17 | 0.26 ± 0.07 | 0.24 ± 0.08 | 0.91 |
|  | **72** | 0.21 ± 0.07 | 0.38 ± 0.23 | 0.31 ± 0.15 | 0.92 | **96** | 0.36 ± 0.15 | 0.31 ± 0.07 | 0.16 ± 0.02 | 0.70 |
| (*E*)-β-farnesene | **0** | 1.69 ± 0.28 | 1.25 ± 0.23 | 1.31 ± 0.28 | 0.56 | **24** | 3.31 ± 0.48 | 5.05 ± 1.35 | 7.80 ± 2.40 | 0.31 |
|  | **24** | 1.77 ± 0.34 | 0.97 ± 0.19 | 1.26 ± 0.26 | 0.18 | **48** | 3.48 ± 0.42 | 5.50 ± 1.54 | 7.62 ± 1.80 | 0.11 |
|  | **48** | 1.49 ± 0.29 | 1.11 ± 0.26 | 1.25 ± 0.24 | 0.61 | **72** | 3.69 ± 0.80 | 5.55 ± 1.73 | 6.57 ± 1.81 | 0.63 |
|  | **72** | 1.67 ± 0.37 | 1.04 ± 0.25 | 1.21 ± 0.27 | 0.37 | **96** | 3.36 ± 0.58 | 4.33 ± 1.19 | 5.44 ± 1.63 | 0.77 |
| β-phellandrene | **0** | 2.5 ± 0.69 | 1.65 ± 0.55 | 1.82 ± 0.43 | 0.68 | **24** | 2.53 ± 0.55 | 3.69 ± 0.81 | 4.09 ± 1.26 | 0.47 |
|  | **24** | 3.91 ± 2.10 | 1.14 ± 0.36 | 1.84 ± 0.62 | 0.36 | **48** | 2.99 ± 0.80 | 3.06 ± 0.61 | 2.21 ± 0.46 | 0.76 |
|  | **48** | 2.67 ± 1.02 | 1.40 ± 0.50 | 1.57 ± 0.52 | 0.79 | **72** | 2.85 ± 0.56 | 4.63 ± 1.63 | 1.89 ± 0.39 | 0.50 |
|  | **72** | 2.94 ± 1.28 | 1.65 ± 0.36 | 1.27 ± 0.46 | 0.67 | **96** | 2.81 ± 0.67 | 4.45 ± 1.90 | 1.93 ± 0.09 | 0.72 |

Supplemental Table S3: Statistical details (Mann-Whitney *U* test) on the analysis of the impact of *Diprion pini* egg deposition on the emission rates of *Pinus sylvestris* key terpenoids that are known to attract the egg parasitoid *Closterocerus ruforum*. Statistical comparison of the emission rates that were recorded by GC-MS analysis of odor from untreated pine (U) and pine with sawfly egg deposition (E) (compare Table S2: U versus U+E). Headspace samples were collected repeatedly from each tree at four different time intervals after the end of the egg deposition period (24, 48, 72, 96 h). Red: *p* values < 0.05.

| Compound | Time point (h) | P-values |
| --- | --- | --- |
| (*E*)-β-ocimene | **24** | 0.94 |
|  | **48** | 0.75 |
|  | **72** | 0.94 |
|  | **96** | 0.28 |
| (*Z*)-β-ocimene | **24** | 0.01 |
|  | **48** | 0.03 |
|  | **72** | 0.02 |
|  | **96** | 0.02 |
| β-caryophyllene | **24** | 0.28 |
|  | **48** | 0.05 |
|  | **72** | 0.09 |
|  | **96** | 0.23 |
| α-humulene | **24** | 0.34 |
|  | **48** | 0.10 |
|  | **72** | 0.62 |
|  | **96** | 0.94 |
| (*E*)-β-farnesene | **24** | 0.01 |
|  | **48** | 0.01 |
|  | **72** | 0.04 |
|  | **96** | 0.12 |
| β-phellandrene | **24** | 0.87 |
|  | **48** | 0.94 |
|  | **72** | 0.43 |
|  | **96** | 0.94 |
